# Supplementary material for: Do wealth and inequality associate with health in a small-scale subsistence society?
Source: eLife. 2021 May 14;10:e59437. doi: 10.7554/eLife.59437 (PMC8225390; doi:10.7554/eLife.59437)
Supplement: Supplementary file 1. — a: Model summary – depression. b: Model summary – social conflicts. c: Model summary – fewer labor partners. d: Model summary – non-social problems. e: Model summary – cortisol. f: Model summary – BMI. g: Model summary – systolic blood pressure. h: Model summary – diastolic blood pressure. i: Model summary – worse self-rated health. j: Model summary – total morbidity. k: Model summary – infections. l: Model summary – respiratory illness. m: Model summary – gastrointestinal illness. n: Gaussian model summaries for juveniles. o: Logistic model summaries for juveniles. p: Overview of exploratory interaction effects. q: Mediation of wealth effects. r: Mediation of inequality effects. s: Mediation of mean community wealth effects. [file elife-59437-supp1.docx]

Table of Contents

[Supplementary file 1a: Model summary – Depression 1](#_Toc65662336)

[Supplementary file 1b: Model summary – Social conflicts 1](#_Toc65662337)

[Supplementary file 1c: Model summary – Fewer Labor partners 1](#_Toc65662338)

[Supplementary file 1d: Model summary – Non-social problems 2](#_Toc65662339)

[Supplementary file 1e: Model summary – Cortisol 2](#_Toc65662340)

[Supplementary file 1f: Model summary – BMI 2](#_Toc65662341)

[Supplementary file 1g: Model summary – Systolic blood pressure 2](#_Toc65662342)

[Supplementary file 1h: Model summary – Diastolic blood pressure 2](#_Toc65662343)

[Supplementary file 1i: Model summary – Worse Self-rated health 4](#_Toc65662344)

[Supplementary file 1j: Model summary – Total morbidity 4](#_Toc65662345)

[Supplementary file 1k: Model summary – Infections 4](#_Toc65662346)

[Supplementary file 1l: Model summary – Respiratory illness 4](#_Toc65662347)

[Supplementary file 1m: Model summary – Gastrointestinal illness 5](#_Toc65662348)

[Supplementary file 1n: Gaussian model summaries for juveniles 5](#_Toc65662349)

[Supplementary file 1o: Logistic model summaries for juveniles 5](#_Toc65662350)

[Supplementary file 1p: Overview of exploratory interaction effects 5](#_Toc65662351)

[Supplementary file 1q: Mediation of wealth effects 6](#_Toc65662352)

[Supplementary file 1r: Mediation of inequality effects 6](#_Toc65662353)

[Supplementary file 1s: Mediation of mean community wealth effects 7](#_Toc65662354)

## Supplementary file 1a: Model summary – Depression

|  | Relative Wealth | | | Absolute Wealth | | | No Covariates | | |
| --- | --- | --- | --- | --- | --- | --- | --- | --- | --- |
| N=670 Variable | Mean | Lower 95% CI | Upper 95%CI | Mean | Lower 95% CI | Upper 95%CI | Mean | Lower 95% CI | Upper 95%CI |
| Intercept | -0.25 | -0.55 | 0.08 | -0.25 | -0.56 | 0.06 | 0.07 | -0.12 | 0.25 |
| Gini | 0.02 | -0.17 | 0.24 | 0.02 | -0.17 | 0.20 | 0.09 | -0.09 | 0.24 |
| Community Relative Wealth a | -0.04 | -0.12 | 0.03 |  |  |  |  |  |  |
| Sample Relative Wealth b |  |  |  | -0.04 | -0.12 | 0.03 | -0.06 | -0.14 | 0.02 |
| Mean Community Wealth c | -0.01 | -0.58 | 0.49 |  |  |  |  |  |  |
| Age | 0.37 | 0.24 | 0.47 | 0.37 | 0.27 | 0.49 |  |  |  |
| Sex=male | -0.50 | -0.62 | -0.37 | -0.50 | -0.62 | -0.38 |  |  |  |
| Household Size | 0.10 | 0.01 | 0.19 | 0.10 | 0.02 | 0.18 |  |  |  |
| Distance to San Borja | -0.11 | -0.31 | 0.07 | -0.11 | -0.28 | 0.07 |  |  |  |
| Community Size | -0.17 | -0.43 | 0.09 | -0.17 | -0.42 | 0.07 |  |  |  |
| sd(Community) N=32 | 0.45 | 0.28 | 0.62 | 0.44 | 0.27 | 0.58 | 0.40 | 0.26 | 0.56 |
| sd(Household) N=323 | 0.27 | 0.07 | 0.42 | 0.27 | 0.09 | 0.42 | 0.34 | 0.17 | 0.49 |
| sd(Individual) N=528 | 0.09 | 0.00 | 0.22 | 0.09 | 0.00 | 0.22 | 0.12 | 0.00 | 0.28 |
| Goodness of fit (R2) | 0.34 | 0.25 | 0.41 | 0.34 | 0.25 | 0.41 | 0.27 | 0.17 | 0.36 |
| a Z-score centered on community mean | | | | | | | | | |
| b Z-score centered on full sample | | | | | | | | | |
| c Mean full sample Z-score for the community | | | | | | | | | |

## Supplementary file 1b: Model summary – Social conflicts

|  | Relative Wealth | | | Absolute Wealth | | | No Covariates | | |
| --- | --- | --- | --- | --- | --- | --- | --- | --- | --- |
| N=401 Variable | Mean | Lower 95% CI | Upper 95%CI | Mean | Lower 95% CI | Upper 95%CI | Mean | Lower 95% CI | Upper 95%CI |
| Intercept | -0.15 | -0.47 | 0.18 | -0.14 | -0.43 | 0.19 | 0.02 | -0.16 | 0.22 |
| Gini | -0.04 | -0.24 | 0.12 | -0.04 | -0.21 | 0.13 | -0.05 | -0.24 | 0.12 |
| Community Relative Wealth a | -0.00 | -0.11 | 0.12 |  |  |  |  |  |  |
| Sample Relative Wealth b |  |  |  | 0.00 | -0.11 | 0.11 | 0.01 | -0.10 | 0.12 |
| Mean Community Wealth c | -0.01 | -0.64 | 0.58 |  |  |  |  |  |  |
| Age | 0.26 | 0.10 | 0.43 | 0.26 | 0.09 | 0.42 |  |  |  |
| Sex=male | -0.09 | -0.27 | 0.08 | -0.09 | -0.28 | 0.08 |  |  |  |
| Household Size | 0.22 | 0.09 | 0.35 | 0.21 | 0.09 | 0.34 |  |  |  |
| Distance to San Borja | 0.16 | -0.04 | 0.36 | 0.15 | -0.01 | 0.33 |  |  |  |
| Community Size | -0.04 | -0.26 | 0.20 | -0.04 | -0.24 | 0.18 |  |  |  |
| sd(Community) N=25 | 0.27 | 0.07 | 0.47 | 0.26 | 0.07 | 0.45 | 0.33 | 0.15 | 0.53 |
| sd(Household) N=203 | 0.43 | 0.23 | 0.61 | 0.44 | 0.27 | 0.60 | 0.48 | 0.31 | 0.65 |
| sd(Individual) N=342 | 0.11 | 0.00 | 0.25 | 0.11 | 0.00 | 0.26 | 0.10 | 0.00 | 0.25 |
| Goodness of fit (R2) | 0.32 | 0.19 | 0.43 | 0.33 | 0.21 | 0.43 | 0.31 | 0.17 | 0.41 |
| a Z-score centered on community mean | | | | | | | | | |
| b Z-score centered on full sample | | | | | | | | | |
| c Mean full sample Z-score for the community | | | | | | | | | |

## Supplementary file 1c: Model summary – Fewer Labor partners

|  | Relative Wealth | | | Absolute Wealth | | | No Covariates | | |
| --- | --- | --- | --- | --- | --- | --- | --- | --- | --- |
| N=399 Variable | Mean | Lower 95% CI | Upper 95%CI | Mean | Lower 95% CI | Upper 95%CI | Mean | Lower 95% CI | Upper 95%CI |
| Intercept | -0.04 | -0.32 | 0.21 | -0.05 | -0.33 | 0.22 | 0.02 | -0.15 | 0.20 |
| Gini | -0.05 | -0.32 | 0.17 | -0.05 | -0.27 | 0.15 | -0.05 | -0.25 | 0.13 |
| Community Relative Wealth a | -0.13 | -0.24 | -0.02 |  |  |  |  |  |  |
| Sample Relative Wealth b |  |  |  | -0.13 | -0.24 | -0.03 | -0.17 | -0.27 | -0.06 |
| Mean Community Wealth c | -0.16 | -0.71 | 0.40 |  |  |  |  |  |  |
| Age | -0.02 | -0.15 | 0.10 | -0.02 | -0.15 | 0.10 |  |  |  |
| Sex=male | 0.01 | -0.06 | 0.08 | 0.01 | -0.06 | 0.08 |  |  |  |
| Household Size | -0.34 | -0.48 | -0.18 | -0.34 | -0.49 | -0.18 |  |  |  |
| Distance to San Borja | -0.05 | -0.26 | 0.14 | -0.05 | -0.22 | 0.13 |  |  |  |
| Community Size | -0.08 | -0.29 | 0.13 | -0.09 | -0.28 | 0.09 |  |  |  |
| sd(Community) N=24 | 0.19 | 0.00 | 0.42 | 0.17 | 0.00 | 0.38 | 0.13 | 0.00 | 0.31 |
| sd(Household) N=181 | 0.94 | 0.84 | 1.05 | 0.94 | 0.83 | 1.05 | 1.00 | 0.89 | 1.11 |
| sd(Individual) N=304 | 0.02 | 0.00 | 0.05 | 0.02 | 0.00 | 0.05 | 0.02 | 0.00 | 0.05 |
| Goodness of fit (R2) | 0.91 | 0.90 | 0.92 | 0.91 | 0.90 | 0.92 | 0.91 | 0.90 | 0.92 |
| a Z-score centered on community mean | | | | | | | | | |
| b Z-score centered on full sample | | | | | | | | | |
| c Mean full sample Z-score for the community | | | | | | | | | |

## Supplementary file 1d: Model summary – Non-social problems

|  | Relative Wealth | | | Absolute Wealth | | | No Covariates | | |
| --- | --- | --- | --- | --- | --- | --- | --- | --- | --- |
| N=398 Variable | Mean | Lower 95% CI | Upper 95%CI | Mean | Lower 95% CI | Upper 95%CI | Mean | Lower 95% CI | Upper 95%CI |
| Intercept | -0.13 | -0.51 | 0.19 | -0.10 | -0.50 | 0.23 | -0.04 | -0.25 | 0.18 |
| Gini | -0.15 | -0.37 | 0.06 | -0.17 | -0.38 | 0.05 | -0.19 | -0.41 | 0.01 |
| Community Relative Wealth a | -0.06 | -0.18 | 0.04 |  |  |  |  |  |  |
| Sample Relative Wealth b |  |  |  | -0.06 | -0.16 | 0.07 | -0.05 | -0.17 | 0.05 |
| Mean Community Wealth c | 0.28 | -0.46 | 0.99 |  |  |  |  |  |  |
| Age | 0.03 | -0.14 | 0.18 | 0.03 | -0.13 | 0.19 |  |  |  |
| Sex=male | -0.15 | -0.33 | 0.01 | -0.15 | -0.33 | 0.02 |  |  |  |
| Household Size | 0.06 | -0.07 | 0.18 | 0.06 | -0.06 | 0.20 |  |  |  |
| Distance to San Borja | 0.05 | -0.19 | 0.28 | 0.11 | -0.10 | 0.31 |  |  |  |
| Community Size | -0.25 | -0.52 | -0.01 | -0.25 | -0.52 | 0.02 |  |  |  |
| sd(Community) N=25 | 0.38 | 0.19 | 0.59 | 0.40 | 0.19 | 0.59 | 0.41 | 0.23 | 0.62 |
| sd(Household) N=201 | 0.42 | 0.27 | 0.61 | 0.42 | 0.23 | 0.58 | 0.42 | 0.25 | 0.59 |
| sd(Individual) N=339 | 0.15 | 0.00 | 0.35 | 0.15 | 0.00 | 0.35 | 0.16 | 0.00 | 0.35 |
| Goodness of fit (R2) | 0.38 | 0.26 | 0.49 | 0.38 | 0.26 | 0.49 | 0.36 | 0.24 | 0.48 |
| a Z-score centered on community mean | | | | | | | | | |
| b Z-score centered on full sample | | | | | | | | | |
| c Mean full sample Z-score for the community | | | | | | | | | |

## Supplementary file 1e: Model summary – Cortisol

|  | Relative Wealth | | | Absolute Wealth | | | No Covariates | | |
| --- | --- | --- | --- | --- | --- | --- | --- | --- | --- |
| N=811 Variable | Mean | Lower 95% CI | Upper 95%CI | Mean | Lower 95% CI | Upper 95%CI | Mean | Lower 95% CI | Upper 95%CI |
| Intercept | 0.50 | 0.30 | 0.66 | 0.50 | 0.32 | 0.68 | 0.06 | -0.07 | 0.21 |
| Gini | 0.02 | -0.12 | 0.18 | 0.01 | -0.12 | 0.14 | 0.00 | -0.14 | 0.15 |
| Community Relative Wealth a | -0.02 | -0.08 | 0.05 |  |  |  |  |  |  |
| Sample Relative Wealth b |  |  |  | -0.02 | -0.08 | 0.05 | -0.03 | -0.09 | 0.05 |
| Mean Community Wealth c | 0.05 | -0.32 | 0.45 |  |  |  |  |  |  |
| Age | -0.12 | -0.22 | -0.04 | -0.12 | -0.21 | -0.03 |  |  |  |
| Sex=male | -0.76 | -0.89 | -0.64 | -0.76 | -0.88 | -0.63 |  |  |  |
| Household Size | 0.05 | -0.02 | 0.11 | 0.05 | -0.02 | 0.12 |  |  |  |
| Distance to San Borja | -0.06 | -0.20 | 0.07 | -0.05 | -0.18 | 0.08 |  |  |  |
| Community Size | -0.09 | -0.24 | 0.08 | -0.08 | -0.23 | 0.06 |  |  |  |
| sd(Community) N=34 | 0.25 | 0.13 | 0.40 | 0.25 | 0.12 | 0.38 | 0.29 | 0.16 | 0.44 |
| sd(Household) N=391 | 0.28 | 0.11 | 0.41 | 0.28 | 0.14 | 0.41 | 0.14 | 0.00 | 0.29 |
| sd(Individual) N=588 | 0.19 | 0.00 | 0.35 | 0.18 | 0.00 | 0.35 | 0.51 | 0.39 | 0.62 |
| Goodness of fit (R2) | 0.35 | 0.28 | 0.43 | 0.35 | 0.27 | 0.43 | 0.35 | 0.25 | 0.44 |
| a Z-score centered on community mean | | | | | | | | | |
| b Z-score centered on full sample | | | | | | | | | |
| c Mean full sample Z-score for the community | | | | | | | | | |

##

## Supplementary file 1f: Model summary – BMI

|  | Relative Wealth | | | Absolute Wealth | | | No Covariates | | |
| --- | --- | --- | --- | --- | --- | --- | --- | --- | --- |
| N=5179 Variable | Mean | Lower 95% CI | Upper 95%CI | Mean | Lower 95% CI | Upper 95%CI | Mean | Lower 95% CI | Upper 95%CI |
| Intercept | 0.16 | 0.05 | 0.28 | 0.16 | 0.05 | 0.28 | 0.20 | 0.12 | 0.29 |
| Gini | 0.01 | -0.06 | 0.09 | 0.00 | -0.07 | 0.08 | 0.03 | -0.04 | 0.10 |
| Community Relative Wealth a | -0.01 | -0.04 | 0.02 |  |  |  |  |  |  |
| Sample Relative Wealth b |  |  |  | -0.01 | -0.04 | 0.02 | -0.01 | -0.04 | 0.02 |
| Mean Community Wealth c | 0.06 | -0.13 | 0.28 |  |  |  |  |  |  |
| Age | 0.08 | 0.03 | 0.14 | 0.08 | 0.03 | 0.14 |  |  |  |
| Sex=male | 0.03 | -0.06 | 0.13 | 0.03 | -0.06 | 0.13 |  |  |  |
| Household Size | 0.03 | -0.02 | 0.08 | 0.03 | -0.02 | 0.08 |  |  |  |
| Distance to San Borja | -0.09 | -0.18 | -0.00 | -0.08 | -0.16 | 0.00 |  |  |  |
| Community Size | 0.03 | -0.07 | 0.13 | 0.03 | -0.07 | 0.14 |  |  |  |
| sd(Community) N=40 | 0.16 | 0.06 | 0.27 | 0.16 | 0.06 | 0.27 | 0.17 | 0.08 | 0.27 |
| sd(Household) N=833 | 0.28 | 0.16 | 0.38 | 0.26 | 0.12 | 0.38 | 0.25 | 0.11 | 0.39 |
| sd(Individual) N=1901 | 1.03 | 0.98 | 1.07 | 1.03 | 0.98 | 1.08 | 1.03 | 0.98 | 1.08 |
| Goodness of fit (R2) | 0.84 | 0.83 | 0.84 | 0.84 | 0.83 | 0.84 | 0.84 | 0.83 | 0.84 |
| a Z-score centered on community mean | | | | | | | | | |
| b Z-score centered on full sample | | | | | | | | | |
| c Mean full sample Z-score for the community | | | | | | | | | |

## Supplementary file 1g: Model summary – Systolic blood pressure

|  | Relative Wealth | | | Absolute Wealth | | | No Covariates | | |
| --- | --- | --- | --- | --- | --- | --- | --- | --- | --- |
| N=3195 Variable | Mean | Lower 95% CI | Upper 95%CI | Mean | Lower 95% CI | Upper 95%CI | Mean | Lower 95% CI | Upper 95%CI |
| Intercept | -0.47 | -0.55 | -0.39 | -0.47 | -0.55 | -0.38 | -0.03 | -0.09 | 0.04 |
| Gini | 0.05 | 0.00 | 0.10 | 0.08 | 0.02 | 0.13 | 0.07 | 0.01 | 0.13 |
| Community Relative Wealth a | -0.01 | -0.05 | 0.03 |  |  |  |  |  |  |
| Sample Relative Wealth b |  |  |  | -0.03 | -0.06 | 0.01 | -0.02 | -0.06 | 0.02 |
| Mean Community Wealth c | -0.29 | -0.43 | -0.16 |  |  |  |  |  |  |
| Age | 0.34 | 0.30 | 0.39 | 0.35 | 0.31 | 0.40 |  |  |  |
| Sex=male | 0.30 | 0.24 | 0.38 | 0.30 | 0.23 | 0.37 |  |  |  |
| Household Size | -0.04 | -0.08 | -0.00 | -0.04 | -0.08 | 0.00 |  |  |  |
| Distance to San Borja | 0.07 | 0.02 | 0.13 | 0.03 | -0.03 | 0.09 |  |  |  |
| Community Size | 0.12 | 0.06 | 0.18 | 0.08 | 0.01 | 0.15 |  |  |  |
| sd(Community) N=40 | 0.07 | 0.00 | 0.14 | 0.11 | 0.02 | 0.20 | 0.14 | 0.06 | 0.21 |
| sd(Household) N=777 | 0.17 | 0.06 | 0.28 | 0.19 | 0.08 | 0.28 | 0.22 | 0.11 | 0.32 |
| sd(Individual) N=1622 | 0.42 | 0.36 | 0.48 | 0.42 | 0.36 | 0.48 | 0.52 | 0.46 | 0.58 |
| Goodness of fit (R2) | 0.34 | 0.31 | 0.38 | 0.34 | 0.31 | 0.38 | 0.34 | 0.30 | 0.38 |
| a Z-score centered on community mean | | | | | | | | | |
| b Z-score centered on full sample | | | | | | | | | |
| c Mean full sample Z-score for the community | | | | | | | | | |

## Supplementary file 1h: Model summary – Diastolic blood pressure

|  | Relative Wealth | | | Absolute Wealth | | | No Covariates | | |
| --- | --- | --- | --- | --- | --- | --- | --- | --- | --- |
| N=3195 Variable | Mean | Lower 95% CI | Upper 95%CI | Mean | Lower 95% CI | Upper 95%CI | Mean | Lower 95% CI | Upper 95%CI |
| Intercept | -0.35 | -0.45 | -0.24 | -0.35 | -0.47 | -0.23 | -0.04 | -0.12 | 0.05 |
| Gini | 0.02 | -0.05 | 0.09 | 0.04 | -0.03 | 0.11 | 0.01 | -0.06 | 0.09 |
| Community Relative Wealth a | -0.04 | -0.08 | -0.00 |  |  |  |  |  |  |
| Sample Relative Wealth b |  |  |  | -0.05 | -0.09 | -0.01 | -0.04 | -0.08 | -0.00 |
| Mean Community Wealth c | -0.21 | -0.38 | -0.02 |  |  |  |  |  |  |
| Age | 0.26 | 0.21 | 0.30 | 0.25 | 0.21 | 0.30 |  |  |  |
| Sex=male | 0.21 | 0.14 | 0.28 | 0.21 | 0.14 | 0.28 |  |  |  |
| Household Size | 0.02 | -0.02 | 0.06 | 0.02 | -0.02 | 0.06 |  |  |  |
| Distance to San Borja | 0.12 | 0.04 | 0.20 | 0.09 | 0.01 | 0.17 |  |  |  |
| Community Size | 0.06 | -0.04 | 0.16 | 0.04 | -0.06 | 0.13 |  |  |  |
| sd(Community) N=40 | 0.18 | 0.11 | 0.25 | 0.19 | 0.12 | 0.27 | 0.20 | 0.14 | 0.28 |
| sd(Household) N=777 | 0.24 | 0.16 | 0.30 | 0.23 | 0.15 | 0.30 | 0.22 | 0.13 | 0.30 |
| sd(Individual) N=1622 | 0.29 | 0.21 | 0.37 | 0.29 | 0.21 | 0.37 | 0.36 | 0.29 | 0.43 |
| Goodness of fit (R2) | 0.24 | 0.20 | 0.28 | 0.24 | 0.20 | 0.28 | 0.23 | 0.19 | 0.27 |
| a Z-score centered on community mean | | | | | | | | | |
| b Z-score centered on full sample | | | | | | | | | |
| c Mean full sample Z-score for the community | | | | | | | | | |

## Supplementary file 1i: Model summary – Worse Self-rated health

|  | Relative Wealth | | | Absolute Wealth | | | No Covariates | | |
| --- | --- | --- | --- | --- | --- | --- | --- | --- | --- |
| N=2523 Variable | Mean | Lower 95% CI | Upper 95%CI | Mean | Lower 95% CI | Upper 95%CI | Mean | Lower 95% CI | Upper 95%CI |
| Intercept | -0.08 | -0.20 | 0.04 | -0.08 | -0.19 | 0.04 | -0.03 | -0.09 | 0.04 |
| Gini | -0.02 | -0.08 | 0.06 | -0.01 | -0.07 | 0.06 | -0.02 | -0.08 | 0.04 |
| Community Relative Wealth a | -0.02 | -0.06 | 0.02 |  |  |  |  |  |  |
| Sample Relative Wealth b |  |  |  | -0.03 | -0.07 | 0.02 | -0.02 | -0.06 | 0.02 |
| Mean Community Wealth c | -0.07 | -0.24 | 0.12 |  |  |  |  |  |  |
| Age | 0.16 | 0.10 | 0.21 | 0.16 | 0.10 | 0.22 |  |  |  |
| Sex=male | -0.26 | -0.34 | -0.17 | -0.26 | -0.34 | -0.17 |  |  |  |
| Household Size | -0.00 | -0.05 | 0.04 | -0.00 | -0.04 | 0.04 |  |  |  |
| Distance to San Borja | 0.03 | -0.05 | 0.11 | 0.03 | -0.05 | 0.09 |  |  |  |
| Community Size | -0.00 | -0.09 | 0.10 | -0.00 | -0.09 | 0.09 |  |  |  |
| sd(Community) N=40 | 0.16 | 0.09 | 0.24 | 0.16 | 0.09 | 0.23 | 0.14 | 0.08 | 0.22 |
| sd(Household) N=689 | 0.07 | 0.00 | 0.16 | 0.07 | 0.00 | 0.16 | 0.07 | 0.00 | 0.16 |
| sd(Individual) N=1307 | 0.37 | 0.29 | 0.44 | 0.36 | 0.28 | 0.44 | 0.39 | 0.31 | 0.46 |
| Goodness of fit (R2) | 0.18 | 0.13 | 0.23 | 0.18 | 0.13 | 0.23 | 0.17 | 0.12 | 0.22 |
| a Z-score centered on community mean | | | | | | | | | |
| b Z-score centered on full sample | | | | | | | | | |
| c Mean full sample Z-score for the community | | | | | | | | | |

## Supplementary file 1j: Model summary – Total morbidity

|  | Relative Wealth | | | Absolute Wealth | | | No Covariates | | |
| --- | --- | --- | --- | --- | --- | --- | --- | --- | --- |
| N=1542 Variable | Mean | Lower 95% CI | Upper 95%CI | Mean | Lower 95% CI | Upper 95%CI | Mean | Lower 95% CI | Upper 95%CI |
| Intercept | 0.20 | -0.08 | 0.46 | 0.20 | -0.06 | 0.45 | 0.38 | 0.17 | 0.57 |
| Gini | -0.07 | -0.28 | 0.15 | -0.06 | -0.24 | 0.13 | -0.12 | -0.29 | 0.08 |
| Community Relative Wealth a | -0.02 | -0.08 | 0.03 |  |  |  |  |  |  |
| Sample Relative Wealth b |  |  |  | -0.02 | -0.08 | 0.03 | -0.01 | -0.06 | 0.04 |
| Mean Community Wealth c | -0.03 | -0.58 | 0.48 |  |  |  |  |  |  |
| Age | 0.30 | 0.24 | 0.36 | 0.30 | 0.24 | 0.36 |  |  |  |
| Sex=male | -0.16 | -0.24 | -0.07 | -0.16 | -0.25 | -0.07 |  |  |  |
| Household Size | 0.01 | -0.05 | 0.07 | 0.01 | -0.05 | 0.07 |  |  |  |
| Distance to San Borja | 0.13 | -0.07 | 0.32 | 0.14 | -0.06 | 0.32 |  |  |  |
| Community Size | -0.05 | -0.32 | 0.22 | -0.06 | -0.29 | 0.20 |  |  |  |
| sd(Community) N=34 | 0.52 | 0.38 | 0.69 | 0.51 | 0.35 | 0.67 | 0.53 | 0.38 | 0.69 |
| sd(Household) N=653 | 0.35 | 0.27 | 0.43 | 0.35 | 0.27 | 0.42 | 0.32 | 0.23 | 0.41 |
| sd(Individual) N=1306 | 0.09 | 0.00 | 0.21 | 0.09 | 0.00 | 0.21 | 0.14 | 0.00 | 0.29 |
| Goodness of fit (R2) | 0.36 | 0.31 | 0.41 | 0.36 | 0.31 | 0.41 | 0.31 | 0.25 | 0.37 |
| a Z-score centered on community mean | | | | | | | | | |
| b Z-score centered on full sample | | | | | | | | | |
| c Mean full sample Z-score for the community | | | | | | | | | |

## Supplementary file 1k: Model summary – Infections

|  | Relative Wealth | | | Absolute Wealth | | | No Covariates | | |
| --- | --- | --- | --- | --- | --- | --- | --- | --- | --- |
| N=1542 Variable | Mean | Lower 95% CI | Upper 95%CI | Mean | Lower 95% CI | Upper 95%CI | Mean | Lower 95% CI | Upper 95%CI |
| Intercept | -1.69 | -2.39 | -0.87 | -1.70 | -2.49 | -0.94 | -1.33 | -1.86 | -0.77 |
| Gini | -0.62 | -1.20 | -0.01 | -0.68 | -1.24 | -0.16 | -0.61 | -1.11 | -0.12 |
| Community Relative Wealth a | -0.06 | -0.24 | 0.11 |  |  |  |  |  |  |
| Sample Relative Wealth b |  |  |  | -0.05 | -0.21 | 0.13 | -0.03 | -0.19 | 0.12 |
| Mean Community Wealth c | 0.21 | -1.00 | 1.44 |  |  |  |  |  |  |
| Age | 0.52 | 0.30 | 0.71 | 0.52 | 0.32 | 0.72 |  |  |  |
| Sex=male | -0.43 | -0.73 | -0.14 | -0.43 | -0.73 | -0.15 |  |  |  |
| Household Size | 0.20 | -0.01 | 0.38 | 0.19 | 0.01 | 0.38 |  |  |  |
| Distance to San Borja | -0.20 | -0.76 | 0.39 | -0.15 | -0.68 | 0.36 |  |  |  |
| Community Size | -0.34 | -1.07 | 0.37 | -0.31 | -0.99 | 0.42 |  |  |  |
| sd(Community) N=34 | 1.49 | 1.01 | 2.06 | 1.49 | 0.98 | 2.10 | 1.38 | 0.96 | 1.89 |
| sd(Household) N=653 | 1.02 | 0.64 | 1.41 | 1.03 | 0.68 | 1.42 | 0.87 | 0.52 | 1.25 |
| sd(Individual) N=1306 | 0.24 | 0.00 | 0.58 | 0.24 | 0.00 | 0.60 | 0.21 | 0.00 | 0.51 |
| Goodness of fit (R2) | 0.32 | 0.26 | 0.38 | 0.32 | 0.26 | 0.38 | 0.28 | 0.21 | 0.33 |
| a Z-score centered on community mean | | | | | | | | | |
| b Z-score centered on full sample | | | | | | | | | |
| c Mean full sample Z-score for the community | | | | | | | | | |
| Note: Model is on the logit scale | | | | | | | | | |

## Supplementary file 1l: Model summary – Respiratory illness

|  | Relative Wealth | | | Absolute Wealth | | | No Covariates | | |
| --- | --- | --- | --- | --- | --- | --- | --- | --- | --- |
| N=1542 Variable | Mean | Lower 95% CI | Upper 95%CI | Mean | Lower 95% CI | Upper 95%CI | Mean | Lower 95% CI | Upper 95%CI |
| Intercept | -1.63 | -2.28 | -1.00 | -1.61 | -2.29 | -1.02 | -1.67 | -2.19 | -1.20 |
| Gini | 0.35 | -0.08 | 0.88 | 0.31 | -0.09 | 0.75 | 0.22 | -0.20 | 0.59 |
| Community Relative Wealth a | -0.04 | -0.20 | 0.12 |  |  |  |  |  |  |
| Sample Relative Wealth b |  |  |  | -0.04 | -0.20 | 0.14 | -0.04 | -0.20 | 0.12 |
| Mean Community Wealth c | 0.16 | -0.97 | 1.20 |  |  |  |  |  |  |
| Age | -0.05 | -0.25 | 0.14 | -0.05 | -0.24 | 0.14 |  |  |  |
| Sex=male | -0.12 | -0.40 | 0.19 | -0.12 | -0.42 | 0.15 |  |  |  |
| Household Size | -0.05 | -0.24 | 0.13 | -0.05 | -0.22 | 0.14 |  |  |  |
| Distance to San Borja | 0.24 | -0.20 | 0.73 | 0.27 | -0.14 | 0.73 |  |  |  |
| Community Size | 0.05 | -0.50 | 0.64 | 0.08 | -0.48 | 0.60 |  |  |  |
| sd(Community) N=34 | 1.08 | 0.66 | 1.60 | 1.06 | 0.66 | 1.56 | 1.01 | 0.60 | 1.46 |
| sd(Household) N=653 | 0.95 | 0.58 | 1.31 | 0.94 | 0.58 | 1.29 | 0.89 | 0.52 | 1.23 |
| sd(Individual) N=1306 | 0.54 | 0.00 | 1.17 | 0.49 | 0.00 | 1.09 | 0.45 | 0.00 | 1.00 |
| Goodness of fit (R2) | 0.22 | 0.14 | 0.33 | 0.21 | 0.13 | 0.32 | 0.20 | 0.12 | 0.30 |
| a Z-score centered on community mean | | | | | | | | | |
| b Z-score centered on full sample | | | | | | | | | |
| c Mean full sample Z-score for the community | | | | | | | | | |
| Note: Model is on the logit scale | | | | | | | | | |

## Supplementary file 1m: Model summary – Gastrointestinal illness

|  | Relative Wealth | | | Absolute Wealth | | | No Covariates | | |
| --- | --- | --- | --- | --- | --- | --- | --- | --- | --- |
| N=1542 Variable | Mean | Lower 95% CI | Upper 95%CI | Mean | Lower 95% CI | Upper 95%CI | Mean | Lower 95% CI | Upper 95%CI |
| Intercept | -0.86 | -1.25 | -0.50 | -0.85 | -1.22 | -0.50 | -0.67 | -0.99 | -0.35 |
| Gini | -0.12 | -0.44 | 0.21 | -0.05 | -0.32 | 0.20 | -0.24 | -0.54 | 0.05 |
| Community Relative Wealth a | -0.01 | -0.12 | 0.13 |  |  |  |  |  |  |
| Sample Relative Wealth b |  |  |  | -0.01 | -0.13 | 0.11 | 0.00 | -0.12 | 0.14 |
| Mean Community Wealth c | -0.32 | -1.07 | 0.45 |  |  |  |  |  |  |
| Age | 0.27 | 0.12 | 0.42 | 0.27 | 0.11 | 0.42 |  |  |  |
| Sex=male | -0.07 | -0.32 | 0.18 | -0.07 | -0.30 | 0.17 |  |  |  |
| Household Size | -0.09 | -0.22 | 0.05 | -0.08 | -0.22 | 0.05 |  |  |  |
| Distance to San Borja | 0.61 | 0.34 | 0.91 | 0.57 | 0.32 | 0.85 |  |  |  |
| Community Size | 0.12 | -0.20 | 0.47 | 0.08 | -0.26 | 0.39 |  |  |  |
| sd(Community) N=34 | 0.61 | 0.36 | 0.88 | 0.59 | 0.36 | 0.84 | 0.78 | 0.50 | 1.06 |
| sd(Household) N=653 | 0.38 | 0.00 | 0.68 | 0.37 | 0.00 | 0.68 | 0.39 | 0.01 | 0.70 |
| sd(Individual) N=1306 | 0.40 | 0.00 | 0.87 | 0.39 | 0.00 | 0.85 | 0.40 | 0.00 | 0.84 |
| Goodness of fit (R2) | 0.16 | 0.10 | 0.25 | 0.16 | 0.10 | 0.24 | 0.14 | 0.08 | 0.23 |
| a Z-score centered on community mean | | | | | | | | | |
| b Z-score centered on full sample | | | | | | | | | |
| c Mean full sample Z-score for the community | | | | | | | | | |
| Note: Model is on the logit scale | | | | | | | | | |

## Supplementary file 1n: Gaussian model summaries for juveniles

|  | BMI | | | Total Morbidity | | |  |
| --- | --- | --- | --- | --- | --- | --- | --- |
| N=4748 Variable | Mean | Lower 95% CI | Upper 95%CI | Mean | Lower 95% CI | Upper 95%CI |  |
| Intercept | 0.05 | -0.09 | 0.20 | -0.13 | -0.37 | 0.11 |  |
| Gini | -0.06 | -0.12 | 0.02 | 0.03 | -0.16 | 0.24 |  |
| Community Relative Wealth a | -0.01 | -0.06 | 0.03 | -0.04 | -0.09 | 0.01 |  |
| Mean Community Wealth c | 0.05 | -0.13 | 0.24 | 0.20 | -0.27 | 0.65 |  |
| Age | 0.01 | -0.13 | 0.14 | 0.14 | -0.04 | 0.29 |  |
| Sex=male | 0.03 | -0.05 | 0.10 | -0.11 | -0.18 | -0.04 |  |
| Household Size | -0.03 | -0.10 | 0.03 | 0.00 | -0.06 | 0.06 |  |
| Distance to San Borja | -0.06 | -0.14 | 0.02 | 0.07 | -0.10 | 0.24 |  |
| Community Size | -0.04 | -0.11 | 0.05 | -0.17 | -0.38 | 0.02 |  |
| sd(Community) N=35 | 0.10 | 0.00 | 0.18 | 0.38 | 0.24 | 0.52 |  |
| sd(Household) N=538 | 0.43 | 0.38 | 0.48 | 0.37 | 0.31 | 0.43 |  |
| sd(Individual) N=1765 | 0.58 | 0.55 | 0.62 | 0.06 | 0.00 | 0.13 |  |
| Goodness of fit (R2) | 0.55 | 0.53 | 0.57 | 0.33 | 0.28 | 0.37 |  |
| a Z-score centered on community mean | | | | | | | |
| b Z-score centered on full sample | | | | | | | |
| c Mean full sample Z-score for the community | | | | | | | |

## Supplementary file 1o: Logistic model summaries for juveniles

|  | Infection | | | Respiratory | | | Gastrointestinal | | |
| --- | --- | --- | --- | --- | --- | --- | --- | --- | --- |
| N=1569 Variable | Mean | Lower 95% CI | Upper 95%CI | Mean | Lower 95% CI | Upper 95%CI | Mean | Lower 95% CI | Upper 95%CI |
| Intercept | -0.37 | -1.38 | 0.63 | -2.39 | -3.16 | -1.61 | -0.97 | -1.73 | -0.22 |
| Gini | -0.23 | -0.87 | 0.44 | 0.21 | -0.29 | 0.68 | 0.17 | -0.36 | 0.66 |
| Community Relative Wealth a | 0.03 | -0.23 | 0.27 | -0.24 | -0.42 | -0.07 | 0.13 | -0.02 | 0.30 |
| Mean Community Wealth c | 0.81 | -0.59 | 2.08 | 0.10 | -0.94 | 1.19 | 0.49 | -0.62 | 1.61 |
| Age | 2.40 | 1.64 | 3.22 | -2.26 | -2.88 | -1.61 | -0.64 | -1.19 | -0.11 |
| Sex=male | -0.65 | -1.07 | -0.22 | -0.11 | -0.40 | 0.16 | 0.06 | -0.17 | 0.34 |
| Household Size | 0.09 | -0.20 | 0.40 | -0.01 | -0.22 | 0.24 | 0.10 | -0.10 | 0.28 |
| Distance to San Borja | 0.04 | -0.58 | 0.70 | 0.11 | -0.36 | 0.55 | 0.40 | -0.06 | 0.95 |
| Community Size | -0.71 | -1.48 | 0.05 | 0.03 | -0.48 | 0.55 | -0.37 | -0.90 | 0.21 |
| sd(Community) N=30 | 1.44 | 0.86 | 2.14 | 0.93 | 0.55 | 1.40 | 1.10 | 0.66 | 1.59 |
| sd(Household) N=469 | 1.26 | 0.89 | 1.73 | 1.14 | 0.86 | 1.45 | 0.85 | 0.60 | 1.14 |
| sd(Individual) N=1423 | 0.33 | 0.00 | 0.82 | 0.48 | 0.00 | 1.07 | 0.36 | 0.00 | 0.85 |
| Goodness of fit (R2) | 0.33 | 0.27 | 0.41 | 0.31 | 0.24 | 0.41 | 0.23 | 0.17 | 0.32 |
| a Z-score centered on community mean | | | | | | | | | |
| b Z-score centered on full sample | | | | | | | | | |
| c Mean full sample Z-score for the community | | | | | | | | | |

## Supplementary file 1p: Overview of exploratory interaction effects

We ran models with all combinations of two way interactions between sex, wealth, and inequality (Sex * Wealth, Sex * Gini, Gini * Wealth) and used the expected log pointwise predictive density (ELPD) to compare model fits. In many cases models were not sufficiently different in ELPD to distinguish, in which case interactions from models with approximately equal predictive density are noted. Interpretations are based on graphical inspection of posterior predictions (see Fig. 6).

| Outcome | Male * Wealth | Male * Gini | Gini * Wealth | Interpretation |
| --- | --- | --- | --- | --- |
| Depression | - |  |  | Wealthier males have lower depression than poor males or females |
| Social conflicts | + |  | - | Poor individuals have more conflicts in unequal places, while wealthier individuals have more conflicts in equal places. |
| Fewer Labor partners |  |  | - | In unequal places, wealthy people have more labor partners; in equal places, poor people have more |
| Non-social problems | + |  |  | Poor women report more non-social conflicts |
| Cortisol |  | + | + | Gini-cortisol association only seen in wealthy men |
| Body Mass Index |  | - |  | Men have increased BMI in equal places, while women have increased BMI in unequal places |
| Systolic blood pressure | - |  |  | Poor men have higher systolic blood pressure |
| Diastolic blood pressure | - | - |  | Poor men have higher diastolic blood pressure |
| Worse Self-rated health | - |  |  | Wealthy men have better self-rated health that poor men and all women |
| Total morbidity |  | + | + | Poor individuals have higher total morbidity in equal places |
| Infections |  |  |  | No interactions |
| Respiratory illness |  | + | + | Wealthy individuals have lower respiratory illness in equal places, higher in unequal places |
| Gastrointestinal illness |  |  |  | No interactions |

## Supplementary file 1q: Mediation of wealth effects

| Dependent | Mediator | N | Wealth Direct Effect  (Path *c*) | Wealth Indirect Effect  (Path *a* + Path *b*) | Wealth Total Effect | Mediator Effect  (Path *b*) | Proportion Mediated |
| --- | --- | --- | --- | --- | --- | --- | --- |
| BMI | Depression | 589 | 0.01 [-0.04;0.05] | 0.00 [-0.00;0.01] | 0.01 [-0.04;0.05] | -0.07 [-0.14;-0.01] | 30% [-69%;129%] |
|  | Conflicts | 364 | 0.00 [-0.05;0.05] | 0.00 [-0.01;0.01] | 0.01 [-0.05;0.06] | -0.07 [-0.13;0.00] | 8% [-81%;98%] |
|  | Fewer Labor Partners | 361 | 0.01 [-0.05;0.05] | -0.01 [-0.03;0.01] | -0.00 [-0.05;0.05] | 0.05 [-0.06;0.15] | 197% [-60%;454%] |
|  | Non-social Problems | 363 | 0.00 [-0.05;0.06] | 0.00 [-0.00;0.01] | 0.01 [-0.05;0.06] | -0.03 [-0.10;0.05] | 20% [-58%;99%] |
|  | Cortisol | 765 | 0.01 [-0.03;0.06] | -0.00 [-0.00;0.00] | 0.01 [-0.03;0.06] | 0.01 [-0.04;0.06] | -1% [-22%;21%] |
| Diastolic Blood Pressure | Depression | 598 | -0.04 [-0.08;0.01] | 0.00 [-0.00;0.01] | -0.03 [-0.08;0.01] | -0.03 [-0.10;0.02] | -2% [-32%;27%] |
|  | Conflicts | 371 | -0.03 [-0.08;0.02] | 0.00 [-0.01;0.01] | -0.03 [-0.07;0.03] | -0.08 [-0.15;-0.00] | -1% [-75%;73%] |
|  | Fewer Labor Partners | 369 | -0.03 [-0.08;0.02] | 0.00 [-0.01;0.01] | -0.03 [-0.08;0.02] | -0.01 [-0.09;0.08] | -2% [-117%;113%] |
|  | Non-social Problems | 370 | -0.03 [-0.08;0.03] | 0.01 [-0.00;0.02] | -0.02 [-0.08;0.03] | -0.08 [-0.15;0.00] | -25% [-174%;123%] |
|  | Cortisol | 685 | -0.04 [-0.09;-0.00] | -0.00 [-0.01;0.00] | -0.05 [-0.09;-0.00] | 0.06 [0.00;0.12] | 1% [-14%;17%] |
| GI Illness | Depression | 278 | 0.02 [-0.04;0.07] | -0.00 [-0.00;0.00] | 0.02 [-0.04;0.07] | 0.01 [-0.05;0.06] | -0% [-28%;27%] |
|  | Conflicts | 89 | -0.00 [-0.11;0.09] | -0.01 [-0.04;0.01] | -0.01 [-0.13;0.08] | 0.11 [0.01;0.22] | 60% [-118%;238%] |
|  | Fewer Labor Partners | 159 | -0.03 [-0.10;0.04] | 0.00 [-0.01;0.02] | -0.03 [-0.10;0.04] | -0.06 [-0.13;0.01] | -5% [-94%;84%] |
|  | Non-social Problems | 89 | -0.00 [-0.10;0.08] | -0.00 [-0.02;0.01] | -0.00 [-0.10;0.08] | 0.01 [-0.08;0.09] | 10% [-57%;78%] |
|  | Cortisol | 143 | 0.01 [-0.05;0.08] | 0.01 [-0.01;0.03] | 0.02 [-0.05;0.09] | -0.13 [-0.19;-0.06] | 35% [-140%;211%] |
| Infectious Illness | Depression | 278 | 0.00 [-0.06;0.06] | 0.00 [-0.00;0.01] | 0.00 [-0.06;0.06] | -0.02 [-0.08;0.04] | 6% [-32%;45%] |
|  | Conflicts | 89 | -0.03 [-0.13;0.08] | 0.00 [-0.01;0.02] | -0.03 [-0.13;0.07] | -0.01 [-0.13;0.10] | -1% [-68%;66%] |
|  | Fewer Labor Partners | 159 | -0.02 [-0.11;0.04] | -0.00 [-0.01;0.01] | -0.02 [-0.10;0.05] | 0.03 [-0.04;0.09] | 2% [-51%;54%] |
|  | Non-social Problems | 89 | -0.03 [-0.12;0.09] | 0.00 [-0.02;0.02] | -0.03 [-0.12;0.09] | -0.01 [-0.11;0.08] | -2% [-74%;70%] |
|  | Cortisol | 143 | -0.02 [-0.09;0.04] | -0.00 [-0.01;0.01] | -0.03 [-0.10;0.03] | 0.04 [-0.01;0.11] | 8% [-59%;76%] |
| Respiratory Illness | Depression | 278 | -0.04 [-0.09;0.01] | -0.00 [-0.00;0.00] | -0.04 [-0.10;0.00] | 0.02 [-0.03;0.07] | 1% [-14%;16%] |
|  | Conflicts | 89 | 0.01 [-0.07;0.14] | -0.00 [-0.02;0.01] | 0.01 [-0.08;0.12] | 0.01 [-0.13;0.12] | -5% [-79%;70%] |
|  | Fewer Labor Partners | 159 | -0.04 [-0.11;0.02] | -0.00 [-0.01;0.01] | -0.05 [-0.11;0.02] | 0.05 [-0.01;0.11] | 3% [-43%;48%] |
|  | Non-social Problems | 89 | 0.01 [-0.09;0.10] | -0.00 [-0.02;0.01] | 0.00 [-0.10;0.10] | 0.01 [-0.08;0.10] | -10% [-89%;69%] |
|  | Cortisol | 143 | -0.06 [-0.12;0.02] | -0.00 [-0.01;0.00] | -0.06 [-0.12;0.01] | 0.04 [-0.02;0.11] | 3% [-26%;33%] |
| Systolic Blood Pressure | Depression | 598 | 0.00 [-0.04;0.05] | 0.01 [-0.00;0.02] | 0.01 [-0.04;0.06] | -0.13 [-0.20;-0.06] | 52% [-115%;219%] |
|  | Conflicts | 371 | 0.01 [-0.04;0.07] | 0.00 [-0.02;0.02] | 0.01 [-0.05;0.06] | -0.16 [-0.24;-0.08] | 5% [-160%;171%] |
|  | Fewer Labor Partners | 369 | 0.01 [-0.04;0.06] | -0.00 [-0.02;0.01] | 0.01 [-0.05;0.06] | 0.02 [-0.07;0.12] | -24% [-192%;143%] |
|  | Non-social Problems | 370 | 0.00 [-0.05;0.05] | 0.01 [-0.00;0.02] | 0.01 [-0.04;0.06] | -0.10 [-0.19;-0.02] | 54% [-126%;233%] |
|  | Cortisol | 685 | -0.02 [-0.07;0.02] | -0.00 [-0.00;0.00] | -0.02 [-0.06;0.03] | 0.04 [-0.02;0.10] | 1% [-31%;33%] |
| Total Morbidity | Depression | 278 | -0.03 [-0.09;0.05] | -0.00 [-0.01;0.01] | -0.03 [-0.09;0.05] | 0.03 [-0.09;0.14] | 2% [-42%;45%] |
|  | Conflicts | 89 | -0.03 [-0.12;0.05] | 0.00 [-0.04;0.04] | -0.03 [-0.12;0.06] | 0.01 [-0.23;0.28] | -1% [-158%;156%] |
|  | Fewer Labor Partners | 159 | -0.05 [-0.13;0.02] | -0.00 [-0.02;0.01] | -0.05 [-0.13;0.03] | 0.05 [-0.08;0.18] | 1% [-60%;62%] |
|  | Non-social Problems | 89 | -0.03 [-0.11;0.05] | -0.01 [-0.06;0.03] | -0.04 [-0.13;0.05] | 0.11 [-0.09;0.31] | 13% [-140%;166%] |
|  | Cortisol | 143 | -0.02 [-0.10;0.05] | -0.00 [-0.02;0.01] | -0.03 [-0.10;0.04] | 0.01 [-0.11;0.12] | 0% [-87%;88%] |
| Worse Self-Rated Health | Depression | 570 | -0.04 [-0.10;0.01] | -0.00 [-0.01;0.00] | -0.05 [-0.10;0.01] | 0.09 [0.01;0.17] | 7% [-26%;40%] |
|  | Conflicts | 368 | -0.05 [-0.11;0.01] | 0.00 [-0.01;0.01] | -0.05 [-0.11;0.01] | -0.03 [-0.13;0.05] | -0% [-24%;24%] |
|  | Fewer Labor Partners | 359 | -0.05 [-0.10;0.01] | 0.00 [-0.01;0.02] | -0.04 [-0.10;0.02] | -0.03 [-0.14;0.08] | -8% [-106%;89%] |
|  | Non-social Problems | 367 | -0.04 [-0.10;0.02] | -0.01 [-0.03;0.00] | -0.06 [-0.11;0.01] | 0.14 [0.05;0.23] | 20% [-41%;81%] |
|  | Cortisol | 643 | -0.04 [-0.09;0.01] | 0.00 [-0.00;0.00] | -0.04 [-0.09;0.01] | 0.04 [-0.03;0.11] | -0% [-18%;18%] |

Mediation models were estimated in *brms* and summarized with *mediation* in the *sjstats* package. To reduce variation between models due to changing samples, the posterior parameter mean and two times the standard deviation of the posterior from the full models (above) were used as priors for the effects of Gini, wealth, and mean wealth. Numbers in brackets are 90% HPDI.

## Supplementary file 1r: Mediation of inequality effects

| Dependent | Mediator | N | Gini Direct Effect  (Path *c*) | Gini Indirect Effect  (Path *a* + Path *b*) | Gini Total Effect | Mediator Effect  (Path *b*) | Proportion Mediated |
| --- | --- | --- | --- | --- | --- | --- | --- |
| BMI | Depression | 589 | 0.06 [-0.02;0.16] | -0.00 [-0.01;0.01] | 0.06 [-0.03;0.15] | -0.07 [-0.14;-0.01] | -0% [-45%;45%] |
|  | Conflicts | 364 | 0.04 [-0.05;0.13] | 0.00 [-0.01;0.02] | 0.04 [-0.05;0.13] | -0.07 [-0.13;0.00] | 1% [-55%;58%] |
|  | Fewer Labor Partners | 361 | 0.02 [-0.08;0.13] | -0.00 [-0.02;0.02] | 0.02 [-0.08;0.13] | 0.05 [-0.06;0.15] | -1% [-70%;68%] |
|  | Non-social Problems | 363 | 0.04 [-0.05;0.14] | 0.00 [-0.01;0.02] | 0.05 [-0.05;0.14] | -0.03 [-0.10;0.05] | 8% [-60%;76%] |
|  | Cortisol | 765 | 0.01 [-0.08;0.10] | 0.00 [-0.00;0.00] | 0.01 [-0.09;0.09] | 0.01 [-0.04;0.06] | 1% [-24%;26%] |
| Diastolic Blood Pressure | Depression | 598 | 0.07 [0.00;0.14] | -0.00 [-0.01;0.01] | 0.06 [-0.00;0.13] | -0.03 [-0.10;0.02] | -1% [-24%;23%] |
|  | Conflicts | 371 | 0.04 [-0.03;0.11] | 0.00 [-0.01;0.02] | 0.04 [-0.03;0.12] | -0.08 [-0.15;-0.00] | 1% [-61%;64%] |
|  | Fewer Labor Partners | 369 | 0.07 [-0.02;0.15] | 0.00 [-0.01;0.01] | 0.07 [-0.02;0.15] | -0.01 [-0.09;0.08] | 0% [-29%;29%] |
|  | Non-social Problems | 370 | 0.04 [-0.04;0.11] | 0.01 [-0.00;0.04] | 0.05 [-0.02;0.12] | -0.08 [-0.15;0.00] | 24% [-103%;151%] |
|  | Cortisol | 685 | 0.00 [-0.06;0.07] | 0.00 [-0.01;0.01] | 0.01 [-0.06;0.07] | 0.06 [0.00;0.12] | 27% [-57%;111%] |
| GI Illness | Depression | 278 | -0.03 [-0.14;0.08] | -0.00 [-0.01;0.01] | -0.03 [-0.14;0.08] | 0.01 [-0.05;0.06] | 0% [-42%;43%] |
|  | Conflicts | 89 | -0.04 [-0.15;0.08] | -0.00 [-0.05;0.05] | -0.04 [-0.15;0.09] | 0.11 [0.01;0.22] | 3% [-165%;170%] |
|  | Fewer Labor Partners | 159 | 0.05 [-0.11;0.19] | 0.01 [-0.01;0.04] | 0.06 [-0.09;0.22] | -0.06 [-0.13;0.01] | 11% [-74%;97%] |
|  | Non-social Problems | 89 | -0.04 [-0.17;0.07] | -0.00 [-0.03;0.03] | -0.05 [-0.17;0.06] | 0.01 [-0.08;0.09] | 1% [-111%;112%] |
|  | Cortisol | 143 | -0.03 [-0.16;0.10] | 0.01 [-0.03;0.04] | -0.02 [-0.16;0.12] | -0.13 [-0.19;-0.06] | -28% [-215%;160%] |
| Infectious Illness | Depression | 278 | -0.11 [-0.22;-0.00] | 0.00 [-0.01;0.01] | -0.11 [-0.22;-0.00] | -0.02 [-0.08;0.04] | -1% [-16%;14%] |
|  | Conflicts | 89 | -0.18 [-0.36;-0.02] | 0.00 [-0.03;0.02] | -0.18 [-0.36;-0.02] | -0.01 [-0.13;0.10] | -0% [-20%;20%] |
|  | Fewer Labor Partners | 159 | -0.14 [-0.33;0.03] | -0.00 [-0.03;0.01] | -0.15 [-0.33;0.04] | 0.03 [-0.04;0.09] | 2% [-21%;25%] |
|  | Non-social Problems | 89 | -0.17 [-0.35;-0.02] | 0.00 [-0.03;0.03] | -0.17 [-0.35;-0.02] | -0.01 [-0.11;0.08] | -0% [-24%;23%] |
|  | Cortisol | 143 | 0.02 [-0.10;0.13] | -0.00 [-0.02;0.01] | 0.01 [-0.11;0.12] | 0.04 [-0.01;0.11] | -8% [-88%;71%] |
| Respiratory Illness | Depression | 278 | 0.05 [-0.02;0.12] | -0.00 [-0.01;0.01] | 0.04 [-0.02;0.11] | 0.02 [-0.03;0.07] | -2% [-46%;43%] |
|  | Conflicts | 89 | 0.06 [-0.04;0.16] | -0.00 [-0.03;0.02] | 0.05 [-0.05;0.16] | 0.01 [-0.13;0.12] | -1% [-91%;90%] |
|  | Fewer Labor Partners | 159 | 0.06 [-0.08;0.19] | -0.01 [-0.03;0.01] | 0.05 [-0.09;0.18] | 0.05 [-0.01;0.11] | -12% [-110%;86%] |
|  | Non-social Problems | 89 | 0.06 [-0.04;0.18] | -0.00 [-0.03;0.03] | 0.06 [-0.04;0.18] | 0.01 [-0.08;0.10] | -3% [-100%;95%] |
|  | Cortisol | 143 | 0.07 [-0.09;0.23] | -0.00 [-0.02;0.01] | 0.07 [-0.10;0.22] | 0.04 [-0.02;0.11] | -2% [-49%;45%] |
| Systolic Blood Pressure | Depression | 598 | 0.05 [-0.01;0.10] | -0.00 [-0.03;0.02] | 0.05 [-0.01;0.11] | -0.13 [-0.20;-0.06] | -7% [-135%;121%] |
|  | Conflicts | 371 | 0.05 [-0.01;0.11] | 0.00 [-0.03;0.03] | 0.06 [-0.02;0.12] | -0.16 [-0.24;-0.08] | 4% [-102%;110%] |
|  | Fewer Labor Partners | 369 | 0.06 [-0.01;0.13] | -0.00 [-0.01;0.01] | 0.06 [-0.01;0.14] | 0.02 [-0.07;0.12] | -0% [-34%;33%] |
|  | Non-social Problems | 370 | 0.05 [-0.02;0.11] | 0.02 [-0.00;0.04] | 0.06 [-0.00;0.13] | -0.10 [-0.19;-0.02] | 24% [-50%;98%] |
|  | Cortisol | 685 | 0.05 [-0.02;0.11] | 0.00 [-0.01;0.01] | 0.05 [-0.02;0.11] | 0.04 [-0.02;0.10] | 2% [-34%;38%] |
| Total Morbidity | Depression | 278 | -0.20 [-0.34;-0.06] | -0.00 [-0.02;0.02] | -0.21 [-0.34;-0.05] | 0.03 [-0.09;0.14] | 0% [-12%;13%] |
|  | Conflicts | 89 | -0.18 [-0.37;0.02] | -0.00 [-0.06;0.05] | -0.18 [-0.40;0.02] | 0.01 [-0.23;0.28] | 0% [-60%;60%] |
|  | Fewer Labor Partners | 159 | -0.12 [-0.31;0.06] | -0.00 [-0.05;0.03] | -0.13 [-0.31;0.06] | 0.05 [-0.08;0.18] | 3% [-49%;55%] |
|  | Non-social Problems | 89 | -0.16 [-0.36;0.03] | -0.01 [-0.10;0.05] | -0.18 [-0.37;0.02] | 0.11 [-0.09;0.31] | 8% [-57%;72%] |
|  | Cortisol | 143 | 0.05 [-0.14;0.28] | 0.00 [-0.02;0.02] | 0.05 [-0.15;0.27] | 0.01 [-0.11;0.12] | 0% [-43%;43%] |
| Worse Self-Rated Health | Depression | 570 | -0.03 [-0.11;0.05] | 0.00 [-0.01;0.03] | -0.02 [-0.11;0.06] | 0.09 [0.01;0.17] | -14% [-141%;113%] |
|  | Conflicts | 368 | -0.02 [-0.12;0.06] | 0.00 [-0.01;0.01] | -0.02 [-0.12;0.06] | -0.03 [-0.13;0.05] | -0% [-56%;56%] |
|  | Fewer Labor Partners | 359 | -0.05 [-0.14;0.05] | -0.00 [-0.02;0.01] | -0.05 [-0.15;0.05] | -0.03 [-0.14;0.08] | 0% [-51%;52%] |
|  | Non-social Problems | 367 | -0.02 [-0.11;0.07] | -0.02 [-0.06;0.01] | -0.04 [-0.14;0.05] | 0.14 [0.05;0.23] | 53% [-173%;279%] |
|  | Cortisol | 643 | 0.02 [-0.06;0.11] | 0.00 [-0.01;0.01] | 0.02 [-0.05;0.12] | 0.04 [-0.03;0.11] | 4% [-47%;56%] |

Mediation models were estimated in *brms* and summarized with *mediation* in the *sjstats* package. To reduce variation between models due to changing samples, the posterior parameter mean and two times the standard deviation of the posterior from the full models (above) were used as priors for the effects of Gini, wealth, and mean wealth. Numbers in brackets are 95% HPDI.

## Supplementary file 1s: Mediation of mean community wealth effects

| Dependent | Mediator | N | Mean Wealth Direct Effect  (Path *c*) | Mean Wealth Indirect Effect  (Path *a* + Path *b*) | Mean Wealth Total Effect | Mediator Effect  (Path *b*) | Proportion Mediated |
| --- | --- | --- | --- | --- | --- | --- | --- |
| BMI | Depression | 589 | 0.01 [-0.21;0.26] | 0.00 [-0.03;0.05] | 0.02 [-0.23;0.25] | -0.07 [-0.14;-0.01] | 12% [-78%;103%] |
|  | Conflicts | 364 | 0.09 [-0.21;0.37] | 0.00 [-0.05;0.05] | 0.09 [-0.20;0.39] | -0.07 [-0.13;0.00] | 1% [-86%;88%] |
|  | Fewer Labor Partners | 361 | -0.02 [-0.30;0.25] | -0.00 [-0.05;0.04] | -0.02 [-0.31;0.24] | 0.05 [-0.06;0.15] | 10% [-65%;85%] |
|  | Other Problems | 363 | 0.09 [-0.22;0.36] | -0.00 [-0.05;0.03] | 0.09 [-0.22;0.35] | -0.03 [-0.10;0.05] | -2% [-71%;66%] |
|  | Cortisol | 765 | 0.16 [-0.09;0.37] | 0.00 [-0.01;0.01] | 0.16 [-0.07;0.38] | 0.01 [-0.04;0.06] | 0% [-13%;13%] |
| Diastolic Blood Pressure | Depression | 598 | -0.42 [-0.60;-0.23] | 0.00 [-0.02;0.03] | -0.42 [-0.60;-0.22] | -0.03 [-0.10;0.02] | -0% [-6%;6%] |
|  | Conflicts | 371 | -0.31 [-0.54;-0.05] | 0.00 [-0.06;0.05] | -0.31 [-0.57;-0.07] | -0.08 [-0.15;-0.00] | -0% [-26%;26%] |
|  | Fewer Labor Partners | 369 | -0.38 [-0.60;-0.13] | 0.00 [-0.02;0.03] | -0.38 [-0.62;-0.15] | -0.01 [-0.09;0.08] | -0% [-8%;8%] |
|  | Other Problems | 370 | -0.29 [-0.52;-0.04] | -0.01 [-0.09;0.04] | -0.31 [-0.56;-0.08] | -0.08 [-0.15;0.00] | 4% [-25%;32%] |
|  | Cortisol | 685 | -0.35 [-0.53;-0.19] | 0.00 [-0.02;0.03] | -0.35 [-0.52;-0.18] | 0.06 [0.00;0.12] | -1% [-8%;7%] |
| GI Illness | Depression | 278 | -0.28 [-0.55;-0.02] | -0.00 [-0.03;0.02] | -0.28 [-0.57;-0.03] | 0.01 [-0.05;0.06] | 0% [-16%;17%] |
|  | Conflicts | 89 | 0.08 [-0.47;0.56] | -0.00 [-0.25;0.22] | 0.06 [-0.54;0.62] | 0.11 [0.01;0.22] | -5% [-194%;184%] |
|  | Fewer Labor Partners | 159 | -0.16 [-0.49;0.18] | 0.01 [-0.03;0.09] | -0.15 [-0.50;0.20] | -0.06 [-0.13;0.01] | -8% [-88%;72%] |
|  | Other Problems | 89 | 0.09 [-0.44;0.57] | -0.00 [-0.10;0.08] | 0.06 [-0.44;0.61] | 0.01 [-0.08;0.09] | -7% [-157%;143%] |
|  | Cortisol | 143 | 0.11 [-0.19;0.44] | 0.00 [-0.08;0.10] | 0.13 [-0.23;0.43] | -0.13 [-0.19;-0.06] | 3% [-110%;116%] |
| Infectious Illness | Depression | 278 | 0.05 [-0.22;0.35] | 0.00 [-0.02;0.04] | 0.06 [-0.24;0.33] | -0.02 [-0.08;0.04] | 5% [-56%;66%] |
|  | Conflicts | 89 | 0.12 [-0.68;0.83] | -0.00 [-0.10;0.12] | 0.11 [-0.68;0.87] | -0.01 [-0.13;0.10] | -0% [-85%;85%] |
|  | Fewer Labor Partners | 159 | 0.12 [-0.26;0.56] | -0.00 [-0.05;0.03] | 0.11 [-0.30;0.53] | 0.03 [-0.04;0.09] | -4% [-55%;47%] |
|  | Other Problems | 89 | 0.09 [-0.60;0.81] | -0.00 [-0.10;0.11] | 0.09 [-0.61;0.82] | -0.01 [-0.11;0.08] | -0% [-75%;75%] |
|  | Cortisol | 143 | -0.08 [-0.35;0.22] | -0.00 [-0.04;0.04] | -0.08 [-0.36;0.21] | 0.04 [-0.01;0.11] | 1% [-60%;61%] |
| Respiratory Illness | Depression | 278 | 0.17 [-0.02;0.35] | -0.00 [-0.04;0.02] | 0.16 [-0.03;0.35] | 0.02 [-0.03;0.07] | -2% [-35%;31%] |
|  | Conflicts | 89 | 0.33 [-0.14;0.81] | 0.00 [-0.09;0.11] | 0.34 [-0.20;0.77] | 0.01 [-0.13;0.12] | 0% [-55%;55%] |
|  | Fewer Labor Partners | 159 | 0.16 [-0.16;0.47] | -0.01 [-0.06;0.04] | 0.14 [-0.16;0.46] | 0.05 [-0.01;0.11] | -6% [-86%;74%] |
|  | Other Problems | 89 | 0.33 [-0.11;0.84] | 0.00 [-0.10;0.09] | 0.33 [-0.21;0.76] | 0.01 [-0.08;0.10] | 0% [-49%;49%] |
|  | Cortisol | 143 | 0.23 [-0.13;0.60] | -0.00 [-0.04;0.04] | 0.23 [-0.13;0.61] | 0.04 [-0.02;0.11] | -0% [-32%;32%] |
| Systolic Blood Pressure | Depression | 598 | -0.40 [-0.57;-0.23] | 0.00 [-0.05;0.07] | -0.39 [-0.58;-0.21] | -0.13 [-0.20;-0.06] | -1% [-18%;16%] |
|  | Conflicts | 371 | -0.28 [-0.49;-0.09] | 0.00 [-0.12;0.11] | -0.28 [-0.51;-0.06] | -0.16 [-0.24;-0.08] | -0% [-55%;55%] |
|  | Fewer Labor Partners | 369 | -0.38 [-0.56;-0.18] | -0.00 [-0.03;0.03] | -0.38 [-0.58;-0.18] | 0.02 [-0.07;0.12] | 0% [-9%;9%] |
|  | Other Problems | 370 | -0.27 [-0.49;-0.08] | -0.02 [-0.11;0.06] | -0.30 [-0.54;-0.09] | -0.10 [-0.19;-0.02] | 6% [-31%;43%] |
|  | Cortisol | 685 | -0.40 [-0.57;-0.23] | 0.00 [-0.02;0.02] | -0.39 [-0.56;-0.22] | 0.04 [-0.02;0.10] | -0% [-6%;5%] |
| Total Morbidity | Depression | 278 | -0.36 [-0.77;0.03] | -0.00 [-0.07;0.05] | -0.37 [-0.80;-0.01] | 0.03 [-0.09;0.14] | 1% [-25%;27%] |
|  | Conflicts | 89 | -0.27 [-0.92;0.42] | 0.00 [-0.24;0.31] | -0.27 [-0.96;0.55] | 0.01 [-0.23;0.28] | -1% [-130%;127%] |
|  | Fewer Labor Partners | 159 | -0.16 [-0.59;0.30] | -0.01 [-0.10;0.06] | -0.18 [-0.63;0.29] | 0.05 [-0.08;0.18] | 3% [-60%;66%] |
|  | Other Problems | 89 | -0.30 [-1.02;0.37] | 0.01 [-0.26;0.33] | -0.29 [-1.04;0.46] | 0.11 [-0.09;0.31] | -3% [-159%;154%] |
|  | Cortisol | 143 | -0.13 [-0.67;0.36] | -0.00 [-0.05;0.04] | -0.13 [-0.62;0.42] | 0.01 [-0.11;0.12] | 0% [-41%;41%] |
| Worse Self-Rated Health | Depression | 570 | -0.04 [-0.24;0.18] | -0.00 [-0.05;0.04] | -0.04 [-0.24;0.19] | 0.09 [0.01;0.17] | 5% [-105%;114%] |
|  | Conflicts | 368 | -0.04 [-0.28;0.22] | 0.00 [-0.03;0.04] | -0.04 [-0.29;0.22] | -0.03 [-0.13;0.05] | -1% [-75%;74%] |
|  | Fewer Labor Partners | 359 | -0.06 [-0.29;0.19] | 0.00 [-0.03;0.04] | -0.06 [-0.30;0.19] | -0.03 [-0.14;0.08] | -1% [-70%;68%] |
|  | Other Problems | 367 | -0.05 [-0.30;0.21] | 0.03 [-0.07;0.15] | -0.02 [-0.31;0.25] | 0.14 [0.05;0.23] | -184% [-404%;35%] |
|  | Cortisol | 643 | -0.16 [-0.35;0.04] | 0.00 [-0.02;0.03] | -0.16 [-0.35;0.05] | 0.04 [-0.03;0.11] | -1% [-28%;25%] |

Mediation models were estimated in *brms* and summarized with *mediation* in the *sjstats* package. To reduce variation between models due to changing samples, the posterior parameter mean and two times the standard deviation of the posterior from the full models (above) were used as priors for the effects of Gini, wealth, and mean wealth. Numbers in brackets are 90% HPDI.
